# Systemic factors of errors in the case identification process of the national routine health information system: A case study of Modified Field Health Services Information System in the Philippines

**DOI:** 10.1186/1472-6963-11-271

**Published:** 2011-10-14

**Authors:** Shinsuke Murai, Leizel P Lagrada, Julita T Gaite, Naruo Uehara

**Affiliations:** 1 Division of International Health (Quality and Health Systems), Graduate School of Medicine, Tohoku University, Sendai, Japan; 2 Health Policy Development and Planning Bureau, Department of Health, Manila, Philippines; 3 Division of Health Management and Planning, Department of Health Policy Science, Tokyo Medical & Dental University, Graduate School of Medical and Dental science, Tokyo, Japan; 4 Provincial Health Office of Palawan, Puerto Princesa, Philippines

## Abstract

**Background:**

The quality of data in national health information systems has been questionable in most developing countries. However, the mechanisms of errors in the case identification process are not fully understood. This study aimed to investigate the mechanisms of errors in the case identification process in the existing routine health information system (RHIS) in the Philippines by measuring the risk of committing errors for health program indicators used in the Field Health Services Information System (FHSIS 1996), and characterizing those indicators accordingly.

**Methods:**

A structured questionnaire on the definitions of 12 selected indicators in the FHSIS was administered to 132 health workers in 14 selected municipalities in the province of Palawan. A proportion of correct answers (difficulty index) and a disparity of two proportions of correct answers between higher and lower scored groups (discrimination index) were calculated, and the patterns of wrong answers for each of the 12 items were abstracted from 113 valid responses.

**Results:**

None of 12 items reached a difficulty index of 1.00. The average difficulty index of 12 items was 0.266 and the discrimination index that showed a significant difference was 0.216 and above. Compared with these two cut-offs, six items showed non-discrimination against lower difficulty indices of 0.035 (4/113) to 0.195 (22/113), two items showed a positive discrimination against lower difficulty indices of 0.142 (16/113) and 0.248 (28/113), and four items showed a positive discrimination against higher difficulty indices of 0.469 (53/113) to 0.673 (76/113).

**Conclusions:**

The results suggest three characteristics of definitions of indicators such as those that are (1) unsupported by the current conditions in the health system, i.e., (a) data are required from a facility that cannot directly generate the data and, (b) definitions of indicators are not consistent with its corresponding program; (2) incomplete or ambiguous, which allow several interpretations; and (3) complete yet easily misunderstood by health workers.

Taking systemic factors into account, the case identification step needs to be reviewed and designed to generate intended data in health information systems.

## Background

Management of local health systems, especially in low-resource settings, requires relevant indicators and quality data from routine health information systems (RHIS) [1]. However, problems have been repeatedly reported on RHIS, such as (a) unreliable data [2,3], (b) incomplete and delayed reports [4,5], (c) putting too much burden on health workers [6,7] and (d) low use of data [8]. This situation hampers evidence-based decision-making, especially in local health systems that depend on data available mostly on RHIS. Although several efforts have been made to improve the performance of RHIS (e.g. computerization of data processing, simplification of definitions of indicators, revision of recording and reporting forms, and re-structuring of health information systems), most developing countries still lack sufficiently strong and effective health information systems [9].

A similar situation is seen in RHIS in the Philippines. The Field Health Services Information System (FHSIS) is a national RHIS that has been operated by the Department of Health (DOH) since 1990 [6]. The system was updated in 1996 to accommodate the use of the system under the devolution [10,11]. The FHSIS 1996 version was expected to solve persistent problems in the FHSIS 1990 version [12]. However, there are still claims that (a) data are unreliable and (b) reports are delayed [13]. DOH-National Epidemiology Center has made continual interventions, such as computerization of data-handling processes, simplification of indicators and revision of standardized forms. Despite these efforts, effective ways to improve the quality of data in FHSIS remain unclear [13].

Quality of data can be compromised in each step of data handling such as case identification, data transmission, data processing and data analysis. In other countries, inconsistencies of data have been observed in each data-handling step in existing health information systems [2,3]. However, the mechanisms of committing errors in those systems are not fully understood, thus limiting further discussion on development of data-handling steps that may help to reduce the occurrence of errors.

Quality of data is compromised, especially in the case identification step, even if data are appropriately handled through the subsequent steps. Although tasks in the case identification step require health workers to understand the definition of each indicator, prior to this, health workers' understanding requires consistent settings of case definitions that assure that eligible cases are appropriately identified when health workers follow the definitions. Otherwise the definition itself may induce health workers to commit errors by systemic reasons. Such causing factors are known as systemic factors of human errors [14].

Improvement opportunities of RHIS can be identified through responses from health workers who actually work on it. Efforts to reflect health workers' response to achieve quality data have been made by assessments of level of understanding. These efforts have pointed to further trainings for health workers. However, this approach alone cannot identify improvement opportunities regarding systemic factors of human errors in RHIS.

In the education field, instructions to reach a given goal or standard are assessed by a combinational use of level and disparity of understanding of items in a criterion-referenced test [15]. RHIS provides instructions of its standard through an operational manual, various trainings and recoding & reporting forms to achieve a full understanding of the standard among health workers to generate intended data in RHIS. This method of assessment of instructions can be applied to the responses of the health workers to RHIS standard to further understand the mechanisms of committing errors in the case identification step of RHIS.

This study aimed to investigate the mechanisms of committing errors in the case identification step in the existing routine health information system (RHIS) by measuring the level and disparity of health workers' understanding of health program indicators used in the Field Health Services Information System (1996 version) in the Philippines, and characterizing those indicators accordingly.

## Methods

### Data Collection

To gauge health workers' understanding of indicators in the FHSIS 1996 version, a structured questionnaire was administered to 132 health workers who were in charge of the case identification step for the first quarter (January, February and March) of 2006 from 14 selected municipalities, 11 from the mainland and three from nearby islands, in the province of Palawan. These 14 municipalities were selected as priority municipalities of the study because they contributed more than 80% of the reported data to the Provincial Health Office for each of the 12 indicators in the first quarter of 2006.

Health workers, including midwives and public health nurses, are responsible for generating data for FHSIS at the field health facilities such as health posts (Barangay Health Station, BHS) and health centers (Rural Health Unit, RHU) operated by the local government units. They record individual cases and identify eligible cases following the definitions of indicators in the official guideline of FHSIS. Cases seen in private health facilities that did not use public health facilities (BHS and RHU) are not supposed to be included in FHSIS. The eligible cases are then counted and reported monthly to the RHU using standardized reporting forms. In the first quarter of 2006, 132 health workers, including three public health nurses, 128 midwives and one medical technologist, prepared monthly reports in 166 out of 216 BHSs and 14 out of 22 RHUs under the Provincial Health Office of Palawan. Some of these health personnel were assigned in more than one health facility in the target municipalities.

The structured questionnaire including 12 items on definition of 12 selected indicators of FHSIS was developed based on (1) document review of FHSIS report to identify regularly-reported indicators; (2) two separate focus group discussions conducted by local program managers, one for 12 public health nurses conducted by a program manager in the Regional Health Office and another for 14 midwives conducted by the Provincial Health Office, from 13 municipalities in the province of Benguet to find out difficulties of health workers in the case identification step; and (3) key informant interviews with program managers in both the Provincial Health Office of Palawan and Benguet to identify indicators that are perceived of doubtful data. The program managers in both distant provinces - Palawan is located in the southwest of Luzon Island, which is closer to Malaysia, and Benguet is located in the mountainous area of Luzon Island - similarly perceived eleven indicators as generating a doubtful report. These eleven indicators included two for maternal health care, two for family planning, three for child health, one for nutrition and three for infectious diseases. An indicator for malaria control has been added because it is not only perceived as a doubtful indicator but the disease is also highly endemic in Palawan. Thus 12 indicators were selected from a list of 78 indicators in different health programs in the services accomplishment component of FHSIS. Table 1 shows the selected 12 indicators and their definitions in the official guideline of FHSIS.

**Table 1:** Definitions of 12 selected indicators on the official guideline of FHSIS (1996)

Selected indicators in FHSIS	Definition on the official guideline of FHSIS
Pregnant women with 3 or more prenatal visits	Pregnant women who had 3 or more pre-natal visits during the month such that at least one visit occurs during the first trimester, one during the second trimester and at least one during the last trimester.

Pregnant given TT2 plus	Pregnant women given TT2 plus (TT2, TT3, TT4, TTL) during the month.

Family Planning: Current users	The number of FP clients who have been carried over from the previous month after deducting the drop outs of the present month and adding the new acceptors of the previous month. Changing clinic, changing method and restart are included under current users.

Family Planning: New acceptors	Clients who were using a contraceptive method for the first time or new to the program.

Severely Underweight Children (6-59 months)	Children 6-59 months old who were found to be severely underweight. Cases identified should only be reported once during the year.

Pneumonia cases seen (0-59 months)	The number of 0-59 months old children seen at the health facility during the month for consultation due to pneumonia. Severe, very severe pneumonia and non-pneumonia are not included in this indicator.

Infant given BCG*	All concerned are hereby requested to report on a monthly and quarterly basis the number of infants given BCG, DPT1, DPT2, DPT3, OPV1, OPV2, OPV3, HepaB1, HepaB2, HepaB3 and Measles vaccine on a per antigen basis.

Fully Immunized Children (9-11 months)	Children from 9-11 months old who have been given BCG, 3 doses of DPT and OPV and measles vaccine. The child is counted FIC as soon as all the required vaccines are administered without waiting for the child to reach 1 year of age.

Rabies: Animal Bite Cases Seen	Person who were bitten by animal (dogs, cats, and others) during the month.

Malaria: Confirmed Cases	Malaria cases identified throughblood smear.

TB symptomatics with sputum examination	Individuals with symptoms compatible to TB who had sputum examination during the month.

New sputum positive cases initiated treatment	New cases found positive through sputum examination and initiated anti-TB treatment. New cases refer to those who have never taken any anti-TB drugs or who have never taken more than one month of anti-TB drugs.

* No definition on the official guildeline of FHSIS. Indicators and definition added after implementation of FHSIS through the Department Circular No. 289 s.2000.

SOURCE: DOH. Modified-FHSIS Guide for Local Chief Executives and Local Health Personnel in Accomplishing Forms for the Health Information System,1996

Twelve items were developed for the 12 selected indicators. The questionnaire is provided as an additional file 1. Nine items asked to identify eligible cases from a list of eligible and ineligible cases. Two items asked for tasks to identify eligible cases on simulated individual records, and one item asked for a task to calculate the simulated number of cases. Choices in each item were developed based on actual individual cases identified on patient records in BHS and RHU in the provinces of Benguet and Palawan.

Among the cases observed in field visits and identified in focus group discussions, some were difficult to judge whether they are eligible or ineligible under the definitions in the official guideline of FHSIS. These doubtful cases were asked the DOH National Epidemiology Center to judge their eligibility.

Practical utility of the items was checked in a pre-test with 26 health workers in the province of Benguet. To ensure applicability of the items to Palawan, each item was reviewed and modified by each health program manager in the Provincial Health Office and a public health nurse in one RHU in Palawan. A pre-test in Palawan was conducted with three midwives in the city health center of Puerto Princesa, Palawan. The city health center was chosen for the pre-test because the FHSIS report was required for the city health center but had an independent reporting line from that of the province. Expressions of items likely to misguide the respondents were corrected based on interviews with respondents immediately after the pre-test.

The language used in the questionnaire was English, the official language of the Philippines, because (1) all official documents are in English, including all the documents related to FHSIS; (2) high school education is in English and (3) the national board exams for midwives and nurses are conducted in English.

To avoid sharing the contents of the questionnaire among the health workers, the questionnaire was administered by the staff of the Provincial Health Office between April and July 2006, when no training was scheduled in the province. All 132 health workers were asked to visit their respective RHU for orientation regarding the study and instructions for filling out the questionnaire.

### Ethical Consideration

The participation of health workers on this study was on a voluntary basis. The name of the health worker was required but such individual information was used only for research purposes and not for individual assessment. The survey was approved by the Provincial Health Officer and all 14 Municipal Health Officers in the targeted areas.

### Measures and Analysis

For each of the 12 items in the questionnaire, a discrimination index and a difficulty index were calculated, and the patterns of wrong answers were abstracted.

The difficulty index, also known as the proportion of respondents who got the correct answer in an item [16], was used to measure the level of understanding of indicators among the health workers. It ranges from 0 to 1; the closer the index to 1, the higher the number of respondents who correctly understand the content being measured by the item. However, the difficulty index alone does not tell whether an item was equally or unequally understood among respondents, and subsequently cannot identify characteristics of an item that may point to an area of instruction. Thus, in addition to the difficulty index, a generalized upper and lower discrimination index was used to measure the disparity in the level of understanding of indicators among the health workers. It calculates the item's ability to discriminate between those who scored high on the total test and those who scored low by subtracting the difficulty index of the lower group from that of the upper group [15]. It ranges from -1 to 1; the closer the index to |1|, the higher the ability of the item to discriminate between those who scored high on the total test and those who scored low. To calculate the discrimination index for each of the 12 items, the respondents who ranked within the top quartile point of the total number of correct answers was considered the upper group and those who ranked within the bottom quartile point of the total number of correct answers was considered the lower group.

Using the discrimination index, each item on the questionnaire is classified into one of three categories: (a) a positively discriminating item, which means that a significantly larger number of respondents in the upper group than in the lower group answered the item correctly, (b) a negatively discriminating item, which means that a significantly larger number of respondents in the lower group than in the upper group answered it correctly, (c) a non-discriminating item, where the percentage of correct answers in the upper group and the lower group were approximately equal.

Difficulty and discrimination indices are typically used in norm-referenced and criterion-referenced tests in the field of education. The norm-referenced test aims to measure the position of the tested individual in a population who took the test, thus an item with a positive discrimination index against a difficulty index within a certain range is preferred according to the test objective [17]. The criterion-referenced test, which was applied in the present study, aims to measure the level of achievement of respondents against given goals or standards, thus an item with non-discrimination against a higher difficulty index is considered a better achievement of instruction measured by the item [18].

Quality of items in criterion-referenced tests is assessed by sensitivity to the corresponding instructions [19]. We applied this method to the assessment of quality of the existing indicators in FHSIS based on health workers' understandings. Since (1) definitions of indicators in FHSIS were used for the criterion of correct answers in the items and (2) distracters of choices in the given items were developed based on actual cases found on patient records, a positive discrimination index here may indicate that the instruction needs to be revised to be more effective for the lower group and non-discrimination against a low difficulty index may indicate that not only instruction but also revision of the setting of indicators itself may need to be reviewed.

Ideally, to ensure the quality of data in the health information system, all health workers must be able to equally identify eligible cases for indicators at the case identification stage, which means that the discrimination index of each item is not significant while its level of difficulty index is high. Assuming the probability of a correct answer in the upper group (*p*_*1*_) and lower group (*p*_*2*_) is equal (*p*_*1*_=*p*_*2 *_= 0.50), the discrimination index (*B*) which gives *P *(*B*) < 0.05 for a two-tailed test of significance was identified by the following formula [15];

$$P\left(B=k\right)=\sum_{U,L}\left[\left(\begin{array}{c} n_{1}\\ U \end{array}\right)\left(\begin{array}{c} n_{2}\\ L \end{array}\right)0.50^{n_{1}+n_{2}}\right]$$

where the sets of values (*U*, *L*) that generate any given *B *= *k *are available by all integral solutions for *U *and *L *of *n*_*2*_*U *- *n*_*1*_*L *= *k*; *U *= 0, 1,..., *n*_*1*_; *L *= 0, 1,..., *n*_*2*__._

All statistical procedures were done with SPSS version 14.0 and Microsoft Excel 2003.

## Results

The response rate of the questionnaire survey was 94.7% (125/132). 113 valid responses (85.6%) were analyzed after excluding 12 incomplete responses. Table 2 shows the relevant profile of the respondents.

**Table 2:** Profile of 113 respondents

Profile of respondents		Number (n = 113)	%
Education	Public Health Nurse	2	1.8
	Midwife	111	98.2
Type of assigned facility	RHU	10	8.8
	BHS	103	91.2
Location of assigned municipality	Mainland	89	78.8
	Nearby island	24	21.2
Source of knowledge on FHSIS	Formal training	60	53.1
	Informal training	48	42.5
	Self learning	5	4.4

### Difficulty index and discrimination index

**Figure 1:**
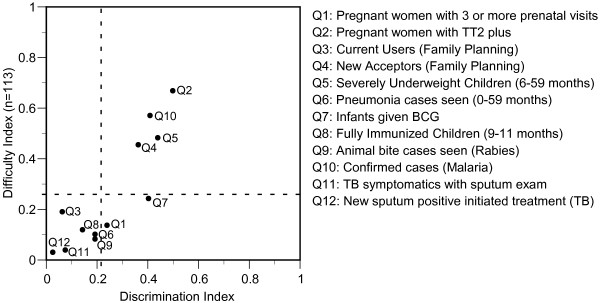
**Difficulty and Discrimination indices for items on 12 selected indicators of FHSIS**. Dotted line parallel to a vertical axis shows the cut-off discrimination index of 0.218 which gives p < 0.05. Dotted line parallel to a horizontal axis shows the average difficulty index of 0.266.

The difficulty index and the discrimination index of each of the 12 items are shown in Figure 1. The discrimination index (*B*) of 0.216 which gives *P *(*B*) < 0.05 for a two-tailed test of significance was determined based on the number of the upper group (*n*_*1 *_= 42) and that of the lower group (*n*_*2 *_= 39). No significant difference was found in the number of the upper group and the lower group by the type of assigned facility (p = 0.203), the location of the assigned municipality (p = 0.783) and the source of FHSIS knowledge (p = 0.756).

None of the items reached a difficulty index of 1.00. An average of the difficulty indices of the 12 items was 0.266. According to the cut-off discrimination index (*B*) of 0.216 and the average difficulty index, 12 items were grouped into three categories (Figure 1). Six items showed non-discrimination against lower difficulty index of 0.035 (4/113) to 0.195 (22/113), two items showed positive discrimination against lower difficulty index of 0.142 (16/113) and 0.248 (28/113) and four items showed positive discrimination against higher difficulty index of 0.469 (53/113) to 0.673 (76/113).

The difficulty index of each item was examined by characteristics of respondents such as an assigned facility, a location of the assigned municipality and a source of FHSIS knowledge. Significant differences were observed on the item on "New acceptors" (p = 0.042) when health workers from RHU (80.0%, 8/10) were compared to those from BHS (42.7%, 44/103), the item on "Fully immunized children (9-11 months)" (p = 0.038) when health workers from mainland municipalities (13.5%, 12/89) were compared to those from island municipalities (41.7%, 10/24), and the item on "TB symptomatic with sputum exam" (p = 0.002) when health workers who received a formal training on FHSIS from the Department of Health or the Provincial Health Office (0%, 0/60) were compared to those who obtained the knowledge of FHSIS through an informal training by a trainer in a municipality who was trained by the Provincial Health Office or the Department of Health (6.3%, 3/48) and those who obtained the knowledge through self-learning (40.0%, 2/5).

### Patterns of wrong answers

An additional file 2 provides 12 items and the frequency of choices in each item.

#### (1) Items with non-discrimination against lower proportion of correct answers

The item on "TB symptomatics with sputum exam" showed a difficulty index of 4.4% (5/113). For this item, 20.4% (23/113) of respondents chose at least all of the eligible cases and added suspected cases of tuberculosis regardless of the results of sputum examinations while the same number of respondents of another 20.4% (23/113) chose only suspected cases of tuberculosis regardless of the results of sputum examinations. Similarly, the item on "TB new sputum positive initiated treatment" showed a difficulty index of 3.5% (4/113). For this item, 37.2% (42/113) of respondents chose at least all of the eligible cases but added the non-eligible case with sputum negative result; 47.8% (54/113) chose some of the eligible cases but failed to choose all of them. The item on "Rabies: Animal bite cases seen" showed a difficulty index of 8.8% (10/113). For this item, 63.7% (72/113) of respondents chose all of the eligible cases but added patients with snake bites under this indicator. The item on "Pneumonia cases seen (0-59 months)" showed a difficulty index of 10.6% (12/113). For this item, 51.3% (58/113) chose at least the eligible case while 38.9% (44/113) added all severe pneumonia cases classified in the case definition of notifiable diseases [12], which were supposed to be excluded under this indicator. Another 15.0% (17/113) chose only the severe pneumonia cases. The item on "Family Planning: Current Users" had a difficulty index of 19.5% (22/113). However, 26.5% (30/113) of respondents answered the number of "Current Users of the reporting month" that was available when the number of "New Acceptor of the reporting month" was used instead of the number of "New Acceptor of the last reporting month" to add to the number of "Current Users of the reporting month" in the calculation. The item on "Fully Immunized Children (9-11 months)" showed a difficulty index of 12.4% (14/113). For this item, 45.1% (51/113) chose at least all of the eligible cases while adding a non-eligible case. For example, 37.2% (42/113) of respondents added children who received the measles vaccine after one year old who were supposed to be excluded.

#### (2) Items with positive discrimination against lower proportion of correct answers

The item on "Pregnant women with 3 or more prenatal visits" showed a difficulty index of 14.2% (16/113). For this item, 64.6% (73/113) of respondents chose at least the eligible case but added the case of a pregnant woman who had her first prenatal visit for the third trimester in the previous month. This same pregnant woman was eligible for the previous month although she had another visit in the third trimester in the current month. The item on "Infant given BCG" had a difficulty index of 24.8% (28/113). For this item, 29.2% (33/113) chose all of the eligible cases but added the non-eligible cases. Another 25.7% (29/113) chose at least an infant given BCG by the assigned facility but excluded those infants who did not reside in the assigned area of the health worker.

#### (3) Items with positive discrimination against higher proportion of correct answers

The item on "Pregnant women with TT2 plus" showed a difficulty index of 67.3% (76/113). However, 17.7% (20/113) of all respondents did not include pregnant women who received the second dose of tetanus toxoid (TT2). The item on "Family Planning: New Acceptors" showed a difficulty index of 46.9% (53/113). For this item, 52.2% (59/113) chose the eligible case but added clients who were supposed to be categorized into Current Users or Drop Outs. For example, 43.4% (49/113) added clients who changed their contraceptive methods and 17.7% (20/113) added clients who changed clinics without changing contraceptive methods. The item on "Severely underweight children (6-59 months)" showed a difficulty index of 48.7% (55/113). For this item, 34.5% (39/113) of respondents chose only either "Severely Underweight" or "Below Normal (Very low)," even though both criteria must be chosen to identify the case. In the malaria control program, the item on "Malaria: Confirmed Cases" showed a difficulty index of 57.5% (65/113). For this item, 17.7% (20/113) of respondents chose at least all of the eligible cases and added suspected cases of malaria regardless of the results of the blood smear examination.

## Discussion

Errors in coding and classification in case identification, such as mistakes in data entry [20] and misinterpretation of the information in the original documents [21], are typically observed as human errors. It is known that human errors in coding and classification tasks have characteristics, such that (1) many errors are non-random [21] and (2) more complex cases are more prone to errors [22]. These characteristics imply underlying unique causes of errors in the case identification step in health information systems.

Regarding the causes of human errors in the case identification step of RHIS, confusion about the definition of indicators among the health workers was reported by the low percentage of respondents who correctly explained the definitions of the indicators [23]. However, lack of training has tended to be considered as the main factor that may cause such confusion but the mechanism of the confusions had been not well-understood.

The present case study of the FHSIS 1996 version in the Philippines demonstrated that indicators can be characterized by how the health workers understand them. The discrimination index and difficulty index for each item show the current understandability of the definition of indicators. When the unique setting of each indicator and pattern of wrong answers are considered, systemic factors behind health workers' confusion are highlighted and their possible countermeasures can be identified.

### Characteristics of indicators

#### (1) Indicators that are unsupported by the current conditions in the health system

Four of six indicators with low difficulty indices that led to non-significant differences in the level of understanding highlight characteristics of indicators that are unsupported by the current conditions in the health system. These indicators can be improved by first ensuring consistency between the definition of the indicator in the FHSIS and the current condition of health systems.

The definition of "TB symptomatics with sputum exam" seems to have been practically simplified by health workers according to the available function of generating data since most of the time health posts were not equipped to do the sputum microscopy. Although this item showed a low difficulty index, those who selected the correct choices may have established a feedback mechanism with their RHUs. Another similar example is "New sputum positive initiated treatment" for tuberculosis. This requires initial treatment but the treatment is given to a patient as soon as the patient is confirmed as tuberculosis at RHU, not at BHS. For both indicators, BHS cannot directly collect data for reporting. Indicators that require data to a facility that cannot directly generate the data was also reported in Chad [24]. On the contrary, the indicator of "Malaria: Confirmed cases" similarly requires blood smear examination at BHS. However it showed a relatively high difficulty index with a positive discrimination index. This can be explained by the availability of barangay malaria microscopists at health posts for blood smear examination in the province of Palawan. Unlike the other malaria endemic areas of the Philippines, Palawan has its own malaria control project called Kilusan Ligtas Malaria that trained and deployed malaria microscopists in the barangays (villages).

There seems to be a gap between the definition and practice of data collection for the indicator on "Rabies: Animal bite cases seen" because animal bite cases seen in RHU and BHS are often transferred to animal bite centers in hospitals and some RHUs, where a post exposure immunization is available and given if necessary. As a result, what health workers know is "animal bite cases transferred" when there was no feedback from animal bite centers. Also, ambiguous definition can be another reason because "others" in the definition of the indicator allows health workers to consider several interpretations. Even "snake bites" was considered as suspected cases of rabies. Since DOH intended "others" to mean animals with risks of transmission of the rabies virus, such as mammals or canines, an additional definition specifying the meaning of "others" would be another approach to make the definition more understandable.

"Pneumonia cases seen (0-59 months)" showed that severe pneumonia cases were considered eligible cases to report while the services accomplishment component of FHSIS excludes severe pneumonia cases from its definition of "Pneumonia cases seen (0-59 months)". This can be partly explained by the inconsistency between the definition in FHSIS and the case definition of notifiable diseases. Although the definition of this indicator in FHSIS covers children aged 0-59 months, there are only two categories for children less than 2 months - severe pneumonia and no pneumonia - according to the case definition of notifiable diseases. Confusions among health workers created by inconsistencies between definition of RHIS and its corresponding program were also known in other countries [25]. Another reason for the confusion can be a limited role of health workers because final diagnosis was often given by doctors while health workers have already counted them as pneumonia cases.

On the contrary, "Severely underweight children (6-59 months)" showed a relatively high difficulty index with a positive discrimination index although there were inconsistencies of two different definitions of weighing in FHSIS and the corresponding program. This indicator had the characteristics of covering cases in both daily health services and biannual special campaigns such as Operation Timbang [26]. In the 2004 update of the nutrition program, a new indicator was introduced for underweight cases reported from special campaigns of Operation Timbang [27], yet there was no update in the corresponding indicator in FHSIS in 2006. Both of these indicators used different criteria following a different standard of growth monitoring. Consequently, both of these criteria must be chosen to identify the eligible case in FHSIS. The health workers with the correct answer seem to have been informed of the situation through the Municipal Nutrition Office since the Nutrition Program gave an explicit instruction of difference between Operation Timbang and FHSIS in the implementation guideline [27].

#### (2) Indicators with incomplete or ambiguous definitions

Two indicators with low difficulty indices that led to significant differences in the level of understanding highlight characteristics of incomplete or ambiguous definitions. These indicators can be improved by clarifying their definitions first.

The item on "Pregnant women with 3 or more prenatal visits" showed that health workers may have at least two other interpretations for the pregnant woman who had several visits in different months in her 3rd trimester, such as reporting the same pregnant woman more than once or reporting the pregnant woman late by waiting until the woman completes the delivery. Even though DOH intended that the eligible pregnant woman be reported once during the pregnancy as soon as she met all criteria in the definition, an explanation of timing and number of reports was lacking in the definition. This would require an additional definition of the timing of report and a definition that the same pregnant woman should be reported only once during her pregnancy.

The item on "Infants given BCG" showed that the definition can allow health workers to consider several interpretations of eligible cases based on different target populations and eligible cases of FHSIS reporting. Since this indicator was added after implementation of the official guideline of FHSIS 1996, the department circular No. 289 s.2000 was distributed to health administrative offices and field health service units to inform them of its definition. Even though DOH intended the case to include infants who received BCG vaccine at field health service units regardless of infants' residence, such as within or outside the catchment area of the assigned field health service unit, there is no explanation of such specification in the definition. This situation allowed another interpretation of reporting infants who received BCG vaccine at the assigned field health service unit only when the infants resided within its catchment area. The original intention of FHSIS was to address the short term data needs of DOH staff managerial or supervisory functions in DOH facilities and in each of the program areas [26]. FHSIS was updated in 1996 to accommodate the situation under the devolution. Thus, Local Government Units were included as users of FHSIS for their management role of field health services unit [11]. These intentions led to a conclusion that the eligible population of all indicators in FHSIS was identified from a population who received health services at the assigned field health service unit. However, identification of eligible cases like an indicator of Fully Immunized Children (9-11 months) requires records of all given antigens even if some of them were not given at the assigned field health service unit. Furthermore, the number of the target population of each of required antigens and Fully Immunized Children (9-11 months) were projected based on total population from census. These practices seem to be a source of the confusion because they imply that eligible cases of reporting in FHSIS are identified within the target population of the responsible field health facility.

#### (3) Indicators with complete definition yet easily misunderstood by health workers

Two indicators with higher difficulty indices that result in a significant difference in the level of understanding among respondents highlight characteristics of indicators with complete definition yet are easily misunderstood by health workers. These indicators may need improved instructions so that all health workers will have the same level of understanding of the indicator. For example, a misunderstanding found in the item on "Pregnant women with TT2 plus" is explained by the understanding of the meaning of "TT2 plus"; "TT2 plus" could be interpreted as more than TT2. The item on "Family Planning: New Acceptors" indicated that most misunderstandings were explained by understanding the meaning of "New Acceptors". "New Acceptors" could suggest "new to methods" or "new to a clinic". Since definitions of these terms are clearly described in the official guideline of FHSIS, these items may show a need for further training. However, specification of terms can be considered further improvement of instructions, such as "TT2 to TT5" instead of "TT2 plus", and "New to program" instead of "New Acceptors". Differences in understanding of "New Acceptors" among health workers in RHU and those in BHS could be partly explained by the existing protocol of the Family Planning Program. Clients who are new to the program are recommended to visit RHU first for an examination, and are then followed up by BHS. Not all clients may visit RHU first but the frequency of meeting such clients will still be higher in RHU.

Even if the definition of the indicator in the FHSIS is consistent with the current condition of health systems, when an item shows a low difficulty index against a non-significant discrimination index, the indicator seems too difficult for health workers and the definition itself may require re-definition to simplify it.

For example, the definitions of the indicators for "Family Planning: Current Users" and "Fully Immunized Children (9-11 months)" were consistent with the current condition of health systems and clearly described in the manual of FHSIS. However, these indicators showed a relatively low difficulty index with a non-significant discrimination index. "Family Planning: Current Users" may be more difficult for health workers even though its definition is clearly described. To fully understand the indicator, health workers need to understand not only the term definitions but also a formula before its actual calculation unlike the other indicators which only require understanding of term definitions.

"Fully immunized children (9-11 months)" demonstrated the confusion among health workers between children who were given all antigens less than 1 year old (Fully Immunized Children) and those given all antigens regardless of being less than 1 year old.

If the instructions do not promote understanding of these indicators, the indicators themselves may require re-definition, such as asking for a report of the components of the formula or introducing a new indicator.

In addition to the systemic reasons described above, weakness in the current mechanism to achieve a full understanding of the FHSIS standard seems to influence the confusions among health workers. For example, the case definition "children given all antigens less than 1 year old" is easy to understand once there is a chance to learn about it. According to staff of the Provincial Health Office of Palawan, it is difficult to keep the right knowledge in their health system because of frequent staff changes and limited opportunities for training. Even though a training of trainers approach has been applied by the Provincial Health Office of Palawan to transfer knowledge and skills to municipalities, a reality of training under municipality level is still unknown. Better understandings of "Fully immunized children (9-11 months)" among health workers in the island municipality may be partly explained by the efficiency of training due to relatively smaller number of health workers. In such a situation under limited opportunities for training, the definition of indicators must be simple, and recording and reporting forms used for routine work need to contain instructions for data handling procedures [28].

Even if these 12 indicators were technically important for the management of local health systems, they are impractical because they risk inducing health workers to commit errors.

**Figure 2:**
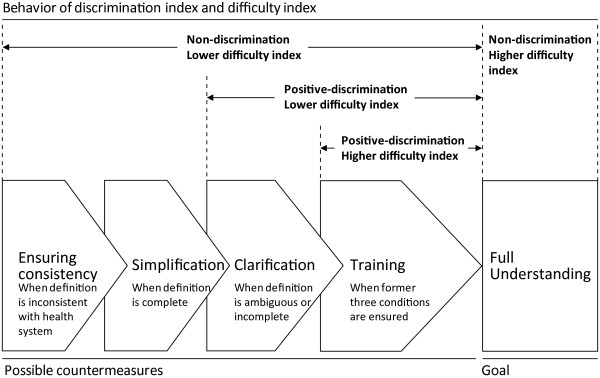
**Behavior of difficulty and discrimination indices and possible countermeasures to be considered**. It provides a guide for a view of investigation of causing factors. For example, when one indicator shows non-discrimination against a low difficulty index, in addition to investigations on ambiguous or incomplete aspect of the definition and a need for further training, investigations for consistency of the definition with the current condition of health system may be required. When the consistency is assured and the definition is found to be complete, an investigation on the difficulty among health workers of the indicator may be required. These investigations are expected to provide the most probable and influential factors for the health workers' confusion.

Figure 2 summarizes a link between behavior of a difficulty index, a discrimination index and possible countermeasures to be considered to achieve full understanding of definition of indicators. It also provides a guide to investigate potential causing factors of errors in case identification. For example, when one indicator shows non-discrimination against low difficulty index, in addition to investigations on ambiguous or incomplete aspect of the definition of indicators and those on needs for further training, investigations may be required for the consistency of the definition with the current condition of health system. Also when the consistency is assured and the definition was found to be complete, an investigation on difficulty among health workers of the indicator may be required. Such investigations are expected to provide the most probable and influencing factors of health workers' confusion. Furthermore, a characteristic of a discrimination index of not independent of the achievement level of the examinee population [29] implies that the combination of discrimination and difficulty indices can be used for tracking the efforts of continuous improvement in the system. For example, with keeping the original intention of the system, when the system design was modified and new instruction was given to health workers, and then health workers' level of understanding was increased, newly calculated discrimination index would tell again newly identified improvement opportunities. Also it would guide investigations into the most probable and influencing factors of health workers' confusions.

In low-information and low-communication technology (ICT) settings, manual and paper-based data-handling are still being used in RHIS, especially in local health systems. In such systems, most data-handling stages depend on human capacity. Even in computerized health information systems, the data entry step cannot be done without human effort. Therefore, control of human errors is one of the key issues to overcome in order to produce quality data in RHIS.

Errors are seen as consequences rather than causes, having their origins in "upstream" systemic factors [14]. The results of our study show that even in an integrated RHIS such as the FHSIS 1996 version in the Philippines, the quality of data for each indicator can be influenced by its characteristics in the case identification stage. More general factors influencing RHIS performance and their hypothetical relationships are described as three determinants, including technical, organizational and behavioral factors [1]. Negative influences of these factors need to be minimized, especially when data-handling of each indicator is done by human effort.

Continuous training is known as an effective method of improving the accuracy of data [30]. However, in low-resource settings, one limitation of a sustainable system operation is the small number of training opportunities [5]. This situation was applicable in our study site in the Philippines. If this is a limitation we could not avoid, the system needs to be designed as simple as possible for health workers while keeping its original purposes. Also, even if enough training opportunities exist, effective training cannot be designed when the standard of the health information system is neither coherent nor continually updated to the current situation of health systems [31]. When the system standard itself contains contradiction or imposes impossible tasks on health workers, we cannot expect that a continuous training approach alone will produce quality data.

In industry, it is well accepted that "quality is built in process [32]". "Process" (a set of interrelated or interacting activities which transform inputs into outputs [33]) is a unit of management that is designed, controlled and improved continuously to produce its intended output. Taking systemic factors into account, the case identification stage needs to be designed to produce its intended output. Further studies are needed of the systemic factors that may affect data quality and process designs that could control them.

Certain limitations of the study should be borne in mind. First, the findings from the questionnaire survey may not be entirely applicable to other areas in Palawan and the Philippines. Data were collected from health workers in municipalities in the mainland and nearby islands of Palawan. These municipalities are more readily accessible to the capital city of Palawan, thus they would be expected to have better access to a formal instruction of FHSIS. Second, the identified possible errors through focus group discussions and field visits, and related systemic factors may be a part of all existing errors and factors, since not all distracters of each indicator may be identified or reflected in the items. Although multiple-answers were applied for asking eligible cases in the questionnaire, difficulty and discrimination indices may change according to the standard of the RHIS and existing cases of distracters in the actual field. Third, the impacts of identified possible errors on quality of data remain unclear. Quality of data may not be influenced even when health workers misunderstood indicators if there was a place with no case of distracters used in each item and the respondent was assigned in such place. Fourth, program assignment, number of years of experience and experience of the specific formal trainings were asked in the questionnaire. However answers were not adequately available. Health workers had difficulty to distinguish the formal training and informal training. Also answers for the assigned program were omitted because the answers were obvious for health workers. In most of the cases, one midwife is assigned for at least one catchment area. Seven health workers refused to provide their year of experience. However, the length of experience did not appear to correlate with the health workers' total score. Coefficient of correlation of year of experience of the available 106 health workers and their total score was -0.063. Fifth, the study focuses on conformance of the current condition to system standard rather than fitness of the standard to use of data. In order to assess the RHIS design in relation with the needs of users, further investigation to gain a better understanding of use of data may be required based on the reality of practices in local health systems. Nevertheless, our results show that indicators can be characterized by how the health workers understand them.

## Conclusions

Taking systemic factors into account, the case identification step needs to be reviewed and designed in order to generate intended data in health information systems.

The present study described three characteristics of definitions of indicators in the case identification step of RHIS such as those that are (1) unsupported by the current conditions in the health system, i.e., (a) data are required from a facility that cannot directly generate the data and, (b) definitions of indicators are not consistent with its corresponding program; (2) incomplete or ambiguous, which allow several interpretations; and (3) complete yet easily misunderstood by health workers. These characteristics highlight the existence of upstream systemic factors that can induce health workers to commit errors.

When attention is given to systemic factors of human errors, health workers' current capability in the case identification step helps to enhance further understanding of improvement opportunities of health information systems. This attention would lead to further discussions of appropriate levels of authorities and concrete countermeasures that could more effectively and efficiently control systemic factors of human errors in RHIS. This implication would also be applicable in developed countries as well as developing countries, where tasks in a case identification step of health information systems depend highly on human effort.

## Feedback to a field

Preliminary findings in Benguet province regarding misinterpretations of the FHSIS guideline were presented at the consultative meeting of FHSIS held in Region-CAR, and reported to DOH-National Epidemiology Center by the Regional FHSIS Coordinator of Region-CAR in 2006. Findings from the present study were given to the Provincial Health Office of Palawan in 2006, 2008 and 2010. Also, taking consideration of identified misunderstandings among health workers, instructions were given to the health workers after the study by the staff of the Provincial Health Office of Palawan. These findings are reflected in the contents of the province's technical advice to health workers.

## Competing interests

The authors declare that they have no competing interests.

## Authors' contributions

SM participated in all phases of the study, from the development of the questionnaire to the preparation of the manuscript, and is the corresponding author. LPL participated in the development of the questionnaire, the interpretation of results, and helped to draft the manuscript. JTG participated in the development of the questionnaire, carried out the data collection and participated in the interpretation of the results. NU participated in the interpretation of the results and helped to draft the manuscript. All authors read and approved the final manuscript.

## Pre-publication history

The pre-publication history for this paper can be accessed here:

http://www.biomedcentral.com/1472-6963/11/271/prepub

## Supplementary Material

Additional file 1 **The questionnaire of understanding of 12 selected indicators of FHSIS**. The additional file 1 is the questionnaire. It contains items on profiles of respondents and the 12 selected indicators. Click here for file

Additional file 2 **Items on 12 selected indicators of FHSIS and frequency of choices by respondents (From Box 1 to Box 12)**. The additional file 2 contains items on 12 selected indicators of FHSIS that were used to gauge the understanding of definitions of indicators among health workers, and the frequency of alternative choices for each of the 12 items. Click here for file
