# Supplementary material for: Systemic factors of errors in the case identification process of the national routine health information system: A case study of Modified Field Health Services Information System in the Philippines
Source: BMC Health Serv Res. 2011 Oct 14;11:271. doi: 10.1186/1472-6963-11-271 (PMC3377923; doi:10.1186/1472-6963-11-271)
Supplement: Additional file 1 — The questionnaire of understanding of 12 selected indicators of FHSIS. The additional file 1 is the questionnaire. It contains items on profiles of respondents and the 12 selected indicators. [file 1472-6963-11-271-S1.PDF]

©Tohoku Univ.

Questionnaire for the personnel who prepares the HIS (FHSIS) Monthly Report

Purpose of this questionnaire

The purpose of this questionnaire is to know how you manage and treat cases which you encounter in your daily activities and how you record them in FHSIS. This is **NOT** to test your skill/ knowledge of Modified-FHSIS.

A case is given for each type of health service that you usually provide to your clients. Please select the choice which reflects your **actual activity**.

If you have not experienced something similar to the case that is given, please select the choice which **you think** it is the most appropriate.

Individual information of respondents is only used for research purpose by Tohoku University.

Individual information

Name:______________________________

Position:__________

Years in the position:___________

Name of RHU/BHS:_____________________

Name of Municipality:____________________

Name of Barangay:______________________

I am assigned for counting the no. of patients/clients for following programs.
(Please check ALL that apply)

☐Maternal Care, ☐Family Planning, ☐EPI, ☐CARI, ☐CDD,
☐Nutrition（Weighing）, ☐Rabies, ☐Malaria, ☐Tuberculosis

Training experience

(1) How did you learn FHSIS? (Please check ONE that mostly apply)

1.____Learn mainly from health staffs through daily work
(Specify if MHO/ PHN/ Midwife ___________)
2.____Learn mainly from self-study with the manual of FHSIS
3.____Learn mainly through training course conducted by DOH or PHO.
4.____Others (Specify) ______________________

(2) Which of the following training/ orientation did you participate?
(Please check ALL that apply)

1.____FHSIS training held by DOH/ PHO within a period from 1989 to 1995
2.____FHSIS training held by DOH/ PHO within a period from 1996 to 2006
3.____Orientation for new employee held by DOH/ PHO (Specify year _______)
4.____Orientation held by PHO in September 2005
5.____Others (Specify the name & year) ______________________

©Tohoku Univ.

Prenatal Care

Given is the TCL for Prenatal Care of your BHS at the end of June, 2005.

Date of Registration (1)	Family Serial Number (2)	Name (3)	Address (4)	Age (5)	LMP/G-P (6)	EDC (7)	Pre-Natal visits (Date) (8)			Risk Code /Date Detected
							First Trimester	Second Trimester	Third Trimester	
10-10-04	×××	AAA	×××	22	9-7-04/G1-P0	6-14-05	10-10-04	1-5-05	4-18-05<br>5-6-05	
12-20-04	×××	BBB	×××	19	9-13-04/G1-P0	6-20-05		12-20-04	4-25-05<br>5-5-05	
10-21-04	×××	CCC	×××	29	9-20-04/G2-P1	6-27-05	10-21-04	1-7-05	6-3-05	
10-24-04	×××	DDD	×××	36	10-4-04/G1-P0	7-11-05	10-24-04<br>(*By BHW)	12-20-04	4-20-05	
10-24-04	×××	EEE	×××	20	10-05-04/G1-P0	7-12-05	10-24-04	2-15-04	4-15-05<br>5-12-05<br>6-1-05	
12-25-04	×××	FFF	×××	21	9-20-04/G1-P0	6-27-05		12-25-04	4-15-05	

Q1.

Which of the following do you consider as “Pregnant women with 3 or more prenatal visits (w/ at least one visit per trimester)" on HIS (FHSIS) Monthly Report of JUNE, 2005? (Please check ALL that apply)

1.____AAA 2.____BBB 3.____CCC 4.____DDD
5.____EEE 6.____FFF

Q2.

Who do you consider as “Pregnant women given TT2 plus” on HIS (FHSIS) Monthly Report? (Please check ALL that apply)

1.____Pregnant women given TT1
2.____Pregnant women given TT1 and TT2
3.____Pregnant women given TT1, TT2 and TT3
4.____Pregnant women given TT1, TT2, TT3 and TT4
5.____Pregnant women given TT1, TT2, TT3, TT4 and TT5

Family Planning

Given is the monthly total number of New Acceptors, Drop Outs and Current Users of **Condom** in **July**.

HIS (FHSIS) Report for the Month: JULY , Year: 2005

FAMILY PLNANING			
METHODS	New Acceptors	Drop Outs	Current Users
Condom	2	3	71

Note: There are no Changing Method, no Changing Clinic, no Transfer and no Restart in July.

Given is the monthly total number of New Acceptors and Drop Outs of **Condom** in **August**.

HIS (FHSIS) Report for the Month: AUGUST , Year: 2005

FAMILY PLNANING			
METHODS	New Acceptors	Drop Outs	Current Users
Condom	6	2	

Note: There are no Changing Method, no Changing Clinic, no Transfer and no Restart in August.
Note: The number of Current Users has not calculated yet.

Q3.

What is the number of **Current Users** of **Condom** for **August**?
The number of Current Users in August is _________.

©Tohoku Univ.

Q4.

Suppose you are identifying the **New Acceptors** of **Condom** in **your facility** to prepare HIS(FHSIS)Monthly Report for July.
Which of the followings do you consider as **New Acceptors** of **Condom** for **your facility in July**? (Please check ALL that apply)

1.____Clients who stopped using Pills in July and started using Condom in July
2.____Clients who started using Condom in July and who are new to Family Planning Program
3.____Clients who have used Condom since June
4.____Clients who have used Condom in another clinic and transferred to your clinic in July and started using Condom in July
5.____Clients who stopped using Condom in another clinic in May and started to use Condom in your clinic in July
6.____Clients who failed to return for a re-supply for Condom
7.____Clients who stopped using Condom in July and started using Pills in July
8.____Clients who transferred out from your clinic and started using Condom in another clinic in July
9.____Clients who stopped using Condom in May and started to use Condom in July

Nutrition

Q5.

Which of the followings do you report as Severely Underweight Children (6-59 months) on HIS (FHSIS) Monthly Report? (Please check ALL that apply)

1.____Children categorized as Severely Underweight
2.____Children categorized as Below Normal (Very Low)
3.____Children categorized as Below Normal (Low)
4.____I do not report Severely Underweight Children (6-59 months) anymore

Child Pneumonia

Q6.

Which of the followings do you report as “Pneumonia cases seen (0-59 months)" on HIS (FHSIS) Monthly Report? (Please check ALL that apply)

1.____Children (2-59 months) with chest indrawing
2.____Children (2-59 months) with fast breathing, fever and cough, but no chest indrawing
3.____Children (2-59 months) with fever and cough, but no chest indrawing and no fast breathing
4.____Children (0-2 months) with chest indrawing or fast breathing
5.____Children (0-2 months) with no severe chest indrawing and no fast breathing

©Tohoku Univ.

EPI

Q7

Which of the following do you report as “infants given BCG” on HIS(FHSIS)Monthly Report? (Please check ALL that apply)

1.____Infant who received BCG at hospital in the month
2.____Infant who received BCG at your facility in the month
3.____Infant who received BCG at somewhere in the other municipality in the month and transferred into your catchment area in the month.
4.____Infant who received BCG at private clinic in your catchment area in the month.
5.____Infant who lives in the other municipality and received BCG at your facility when he/she visited his/her relatives living in your catchment area.

Q8.

Given is Target Group List of EPI at the end of **July**, 2005 (See Attached TCL for EPI), which of the followings do you report as “Fully Immunized Children (9-11 months)" on HIS (FHSIS) Monthly Report of **July**, 2005? (Please check ALL that apply)

1.___A, 2.___B, 3.___C, 4.___D, 5.___E, 6.___F, 7.___G, 8.___H, 9.___J

Rabies

Q9.

Which of the following cases do you report as “Animal bites cases seen” on HIS (FHSIS) Monthly Report? (Please check ALL that apply)

1.____Dog bite cases
2.____Cat bite cases
3.____Snake bite cases
4.____Mosquito bite cases
5.____Monkey bite cases

©Tohoku Univ.

Malaria

Q10.

Which of the followings cases do you report as "Malaria: Confirmed" on HIS (FHSIS) Monthly Report? (Please check ALL that apply)

1.____Those whose blood smear was examined through microscopy and reported as Malaria at laboratory in RHU
2.____Those who are tested by barangay microscopist and confirmed as Malaria.
3.____Those who are clinically diagnosed as Malaria
4.____Those who visited your facility for Mosquito bites

Tuberculosis

Q11.

Which of the following cases do you report as “TB symptomatics with sputum exam” on HIS (FHSIS) Monthly Report? (Please check ALL that apply)

1.____Patient who is complaining of cough with more than 2 weeks
2.____Patient who is complaining of cough with more than 2 weeks and who was given advice by midwife to visit RHU/MHC
3.____Patient who is complaining of cough with more than 2 weeks and whose sputum was collected by midwife (but the sputum has not been sent to RHU yet)
4.____Patient who is complaining of cough with more than 2 weeks and whose sputum was collected by midwife and sent to RHU
5.____Patient with smear (+) result from the laboratory in RHU
6.____Patient with smear (－) result from the laboratory in RHU
7.____Patient with doubtful result from the laboratory in RHU

©Tohoku Univ.

Q12.

Which of the following do you report as “New sputum (+) initiated treatment on HIS (FHSIS) Monthly Report? (Please check ALL that apply)

1.____Patient whose two sputum smear results are positive in the first set of specimens, and has started treatment.
2.____Patient whose one sputum smear result is positive in the first set of specimens, and has started treatment.
3.____Patient whose three sputum smear results are positive in the first set of specimens, and has started treatment.
4.____Patient whose one sputum smear result is positive in the second set of specimens, and has started treatment.

THANK YOU FOR YOUR COOPERATION

(c) Tohoku Univ.

Target Group List for EPI at ***the end of July, 2005***

Date of Birth (1)	Family Serial Number (2)	Name of Infant (3)	Name of Mother (4)	Address (5)	Month Reaches Age1 (6)	Date Fully Immunized (7)	Date Immunization Received (8)											Remarks (9)
							BCG	DPT1	DPT2	DPT3	Polio1	Polio2	Polio3	Measles	Hepa B1	Hepa B2	Hepa B3	
7/20/04	××	AAA	××××	×××	July		7/29/04	9/10/04	10/15/04	11/5/04	9/10/04	10/15/04	11/5/04	7/23/05				
7/20/04	××	BBB	××××	×××	July		7/29/04	9/10/04	10/15/04	11/5/04	9/10/04	10/15/04	11/5/04					
8/20/04	××	CCC	××××	×××	August		8/20/04<br>(Given in Hospitall)	10/8/04	11/12/04	12/17/04	10/8/04	11/12/04	12/17/04	7/14/05				BCG at Hospital
8/20/04	××	DDD	××××	×××	August		8/20/04<br>Given in another BHS in another Municipality	10/8/04	11/12/04	12/17/04	10/8/04	11/12/04	12/17/04	7/14/05				Transferred in
7/20/04	××	EEE	××××	×××	July		7/29/04	9/10/04	10/15/04	11/5/04	9/10/04	10/15/04	11/5/04					
7/29/04	××	FFF	××××	×××	July		8/6/04	9/10/04	10/15/04	11/5/04	9/10/04	10/15/04	11/5/04	6/23/05	9/10/04			
8/15/04	××	GGG	××××	×××	August		8/20/04	10/1/04	11/5/04	12/10/04	10/1/04	11/5/04	12/10/04	7/14/05	10/8/04	11/12/04	12/17/03	
8/20/04	××	HHH	××××	×××	August		8/20/04	10/8/04	11/12/04	12/17/04	10/8/04	11/12/04	12/17/04	7/14/05				
8/20/04	××	JJJ	××××	×××	August		8/20/04	10/8/04	11/12/04	12/17/04	10/8/04	11/12/04	12/17/04					
