# Supplementary material for: Systemic factors of errors in the case identification process of the national routine health information system: A case study of Modified Field Health Services Information System in the Philippines
Source: BMC Health Serv Res. 2011 Oct 14;11:271. doi: 10.1186/1472-6963-11-271 (PMC3377923; doi:10.1186/1472-6963-11-271)
Supplement: Additional file 2 — Items on 12 selected indicators of FHSIS and frequency of choices by respondents (From Box 1 to Box 12). The additional file 2 contains items on 12 selected indicators of FHSIS that were used to gauge the understanding of definitions of indicators among health workers, and the frequency of alternative choices for each of the 12 items. [file 1472-6963-11-271-S2.PDF]

Box 1. TB symptomatics with sputum exam (Q11)

Which of the following cases do you report as “TB symptomatic with sputum exam” on HIS (FHSIS) Monthly Report? (Please check ALL that apply)

Choices given	Frequency	% (n=113)
Patient who is complaining of cough with more than 2 weeks	42	37.2
Patient who is complaining of cough with more than 2 weeks and who was given advice by midwife to visit RHU/MHC.	44	38.9
Patient who is complaining of cough with more than 2 weeks and whose sputum was collected by midwife (but the sputum has not been sent to RHU yet)	41	36.3
Patient who is complaining of cough with more than 2 weeks and whose sputum was collected by midwife and sent to RHU	79	69.9
* Patient with smear (+) result from the laboratory in RHU	74	65.5
* Patient with smear (—) result from the laboratory in RHU	54	47.8
* Patient with doubtful result from the laboratory in RHU	33	29.2

*Correct choice

Box 2. New sputum (+) initiated treatment (Q12)

Which of the following do you report as “New sputum (+) initiated treatment on HIS (FHSIS) Monthly Report? (Please check ALL that apply)

Choices given	Frequency	% (n=113)
* Patient whose two sputum smear results are positive in the first set of specimens, and has started treatment.	81	71.7
Patient whose one sputum smear result is positive in the first set of specimens, and has started treatment.	55	48.7
* Patient whose three sputum smear results are positive in the first set of specimens, and has started treatment.	103	91.2
* Patient whose one sputum smear result is positive in the second set of specimens, and has started treatment.	49	43.4

*Correct choice

Box 3. Malaria: Confirmed Cases (Q10)

Which of the followings cases do you report as "Malaria: Confirmed" on HIS (FHSIS) Monthly Report? (Please check ALL that apply)

Choices given	Frequency	% (n=113)
* Those whose blood smear was examined through microscopy and reported as Malaria at laboratory in RHU.	100	88.5
* Those who are tested by barangay microscopist and confirmed as Malaria.	97	85.8
Those who are clinically diagnosed as Malaria.	21	18.6
Those who visited your facility for Mosquito bites.	2	1.8

*Correct choice

Box 4. “Severely Underweight Children (6-59 months)” (Q5)

Which of the followings do you report as Severely Underweight Children (6-59 months) on HIS (FHSIS) Monthly Report? (Please check ALL that apply)

Choices given	Frequency	% (n=113)
* Children categorized as Severely Underweight	93	82.3
* Children categorized as Below Normal (Very Low)	83	73.5
Children categorized as Below Normal (Low)	16	14.2
I do not report Severely Underweight Children (6-59 months) anymore	7	6.2

*Correct choice

Box 5. “Pneumonia cases seen (0-59 months)” (Q6)

Which of the followings do you report as “Pneumonia cases seen (0-59 months)" on HIS (FHSIS) Monthly Report? (Please check ALL that apply)

Choices given	Frequency	% (n=113)
Children (2-59 months) with chest indrawing	74	65.5
* Children (2-59 months) with fast breathing, fever and cough, but no chest indrawing	70	61.9
Children (2-59 months) with fever and cough, but no chest indrawing and no fast breathing	6	5.3
Children (0-2 months) with chest indrawing or fast breathing	93	82.3
Children (0-2 months) with no severe chest indrawing and no fast breathing	14	12.4

*Correct choice

Box 6. “Pregnant women with 3 or more prenatal visits” (Q1)

Given is the TCL for Prenatal Care of your BHS at the end of June, 2005.

Date of Registration (1)	Family Serial Number (2)	Name (3)	Address (4)	Age (5)	LMP/G-P (6)	EDC (7)	Pre-Natal visits (Date) (8)			Risk Code /Date Detected
							First Trimester	Second Trimester	Third Trimester	
10-10-04	× × ×	AAA	× × ×	22	9-7-04/G1-P0	6-14-05	10-10-04	1-5-05	4-18-05<br>5-6-05	
12-20-04	× × ×	BBB	× × ×	19	9-13-04/G1-P0	6-20-05		12-20-04	4-25-05<br>5-5-05	
10-21-04	× × ×	CCC	× × ×	29	9-20-04/G2-P1	6-27-05	10-21-04	1-7-05	6-3-05	
10-24-04	× × ×	DDD	× × ×	36	10-4-04/G1-P0	7-11-05	10-24-04<br>(*By BHW)	12-20-04	4-20-05	
10-24-04	× × ×	EEE	× × ×	20	10-05-04/G1-P0	7-12-05	10-24-04	2-15-04	4-15-05<br>5-12-05<br>6-1-05	
12-25-04	× × ×	FFF	× × ×	21	9-20-04/G1-P0	6-27-05		12-25-04	4-15-05	

Which of the following do you consider as “Pregnant women with 3 or more prenatal visits (w/ at least one visit per trimester)" on HIS (FHSIS) Monthly Report of JUNE, 2005? (Please check ALL that apply)

Choices given	Frequency	% (n=113)
AAA	40	35.4
BBB	3	2.7
* CCC	99	87.6
DDD	16	14.2
EEE	83	73.5
FFF	2	1.8

*Correct choice

Box 7. “Rabies: Animal bite cases seen” (Q9)

Which of the following cases do you report as “Animal bites cases seen” on HIS (FHSIS) Monthly Report? (Please check ALL that apply)

Choices given	Frequency	% (n=113)
* Dog bite cases	112	99.1
* Cat bite cases	100	88.5
Snake bite cases	83	74.5
Mosquito bite cases	5	4.4
* Monkey bite cases	87	77.0

*Correct choice

Box 8. “Infant given BCG” (Q7)

Which of the following do you report as “infants given BCG” on HIS(FHSIS)Monthly Report? (Please check ALL that apply)

Choices given	Frequency	% (n=113)
Infant who received BCG at hospital in the month	39	34.5
* Infant who received BCG at your facility in the month	110	97.3
Infant who received BCG at somewhere in the other municipality in the month and transferred into your catchment area in the month	29	25.7
Infant who received BCG at private clinic in your catchment area in the month	44	38.9
* Infant who lives in the other municipality and received BCG at your facility when he/she visited his/her relatives living in your catchment area	63	55.8

*Correct choice

Box 9. “Pregnant women with TT2 plus” (Q2)

Who do you consider as “Pregnant women given TT2 plus” on HIS (FHSIS) Monthly Report? (Please check ALL that apply)

Choices given	Frequency	% (n=113)
Pregnant women given TT1	7	6.2
* Pregnant women given TT1 and TT2	90	79.6
* Pregnant women given TT1, TT2 and TT3	105	92.9
* Pregnant women given TT1, TT2, TT3 and TT4	102	90.3
* Pregnant women given TT1, TT2, TT3, TT4 and TT5	99	87.6

*Correct choice

Box 10. “Family Planning: New Acceptors” (Q4)

Suppose you are identifying the New Acceptors of Condom in your facility to prepare HIS(FHSIS)Monthly Report for July.

Which of the followings do you consider as New Acceptors of Condom for your facility in July? (Please check ALL that apply)

Choices given	Frequency	% (n=113)
Clients who stopped using Pills in July and started using Condom in July	51	45.1
* Clients who started using Condom in July and who are new to Family Planning Program	111	98.2
Clients who have used Condom since June	6	5.3
Clients who have used Condom in another clinic and transferred to your clinic in July and started using Condom in July	21	18.6
Clients who stopped using Condom in another clinic in May and started to use Condom in your clinic in July	27	23.9
Clients who failed to return for a re-supply for Condom	4	3.5
Clients who stopped using Condom in July and started using Pills in July	2	1.8
Clients who transferred out from your clinic and started using Condom in another clinic in July	11	9.7
Clients who stopped using Condom in May and started to use Condom in July	22	19.5

*Correct choice

Box 11. "Family Planning: Current Users" (Q3)

Given is the monthly total number of New Acceptors, Drop Outs and Current Users of Condom in July.

HIS (FHSIS) Report for the Month: JULY , Year: 2005

FAMILY PLNANING			
METHODS	New Acceptors	Drop Outs	Current Users
Condom	2	3	71

Note: There are no Changing Method, no Changing Clinic, no Transfer and no Restart in July.

Given is the monthly total number of New Acceptors and Drop Outs of **Condom** in **August**.

HIS (FHSIS) Report for the Month: AUGUST , Year: 2005

FAMILY PLNANING			
METHODS	New Acceptors	Drop Outs	Current Users
Condom	6	2	

Note: There are no Changing Method, no Changing Clinic, no Transfer and no Restart in August.
Note: The number of Current Users has not calculated yet.

What is the number of Current Users of Condom for August?

The number of Current Users in August is _________.

	Answers	Frequency	% (n=113)
	0	2	1.8
	4	8	7.1
	68	4	3.5
	69	1	0.9
	70	13	11.5
*	71	22	19.5
	73	11	9.7
	74	16	14.2
	75	30	26.5
	76	1	0.9
	77	3	2.7
	79	1	0.9
	8	1	0.9

*Correct answer

Box 12. “Fully Immunized Children (9-11 months)” (Q8)

Given is Target Group List of EPI at the end of **July**, 2005, which of the followings do you report as “Fully Immunized Children (9-11 months)" on HIS (FHSIS) Monthly Report of **July**, 2005? (Please check ALL that apply)

Date of Birth (1)	Family Serial Number (2)	Name of Infant (3)	Name of Mother (4)	Address (5)	Month Reaches Age 1 (6)	Date Fully Immunized (7)	Date Immunization Received (8)											Remarks (9)
							BCG	DPT1	DPT2	DPT3	Polio1	Polio2	Polio3	Measles	Hepa B1	Hepa B2	Hepa B3	
7/20/04	××	AAA	××××	×××	July		7/29/04	9/10/04	10/15/04	11/5/04	9/10/04	10/15/04	11/5/04	7/23/05				
7/20/04	××	BBB	××××	×××	July		7/29/04	9/10/04	10/15/04	11/5/04	9/10/04	10/15/04	11/5/04					
8/20/04	××	CCC	××××	×××	August		8/20/04 (Given in Hospital)	10/8/04	11/12/04	12/17/04	10/8/04	11/12/04	12/17/04	7/14/05				BCG at Hospital
8/20/04	××	DDD	××××	×××	August		8/20/04<br>Given in another BHS in another Municipality	10/8/04	11/12/04	12/17/04	10/8/04	11/12/04	12/17/04	7/14/05				Transferred in
7/20/04	××	EEE	××××	×××	July		7/29/04	9/10/04	10/15/04	11/5/04	9/10/04	10/15/04	11/5/04					
7/29/04	××	FFF	××××	×××	July		8/6/04	9/10/04	10/15/04	11/5/04	9/10/04	10/15/04	11/5/04	6/23/05	9/10/04			
8/15/04	××	GGG	××××	×××	August		8/20/04	10/1/04	11/5/04	12/10/04	10/1/04	11/5/04	12/10/04	7/14/05	10/8/04	11/12/04	12/17/03	
8/20/04	××	HHH	××××	×××	August		8/20/04	10/8/04	11/12/04	12/17/04	10/8/04	11/12/04	12/17/04	7/14/05				
8/20/04	××	JJJ	××××	×××	August		8/20/04	10/8/04	11/12/04	12/17/04	10/8/04	11/12/04	12/17/04					

Choices given	Frequency	% (n=113)
AAA	65	57.5
BBB	12	10.6
* CCC	86	76.1
* DDD	81	71.7
EEE	14	12.4
FFF	33	29.2
* GGG	89	78.8
* HHH	88	77.9
JJJ	8	7.1

*Correct choice
